# Teaching Antenatal Counseling Skills via Video Conference

**DOI:** 10.7759/cureus.17030

**Published:** 2021-08-09

**Authors:** Amanda J Kim, Rachel Umoren, Megan M Gray

**Affiliations:** 1 Pediatrics/Neonatal-Perinatal Medicine, Oregon Health and Science University, Portland, USA; 2 Neonatology, Seattle Children's Hospital/University of Washington School of Medicine, Seattle, USA

**Keywords:** antenatal counseling, simulation in medical education, skills and simulation training, virtual patient simulations, virtual workshop, video conference

## Abstract

Neonatologists provide counseling to expectant parents to prepare them for the birth and subsequent medical care that their extremely preterm, or otherwise medically complex newborn may require. The skills required to conduct these sensitive conversations are often taught to neonatology trainees via direct observation or simulated scenarios in advance of counseling actual patients. This technical report details how we taught antenatal counseling skills to junior neonatal-perinatal medicine (NPM) fellows via video conferencing during the coronavirus disease 2019 (COVID-19) pandemic. This approach could be used to effectively prepare future trainees to perform antenatal counseling.

## Introduction

Approximately 10% of babies in the United States are born prematurely each year and many additional babies have congenital anomalies or birth defects [1]. The majority of these babies will require care in a neonatal intensive care unit (NICU). Antenatal counseling is an important task performed by neonatologists to prepare expectant parents for the experience of having their baby receive NICU care. Neonatologists provide information and facilitate discussions between family members and the medical team to support shared decision-making during the extremely stressful events of preterm labor or the prenatal diagnosis of congenital anomalies. While these conversations are difficult, they are also the first chance to build trust and develop a therapeutic relationship with a family whose baby may receive NICU care for weeks or months. Therefore, it is critical that neonatal clinicians have advanced communication skills and training to respond to emotions likely to be elicited from families during antenatal counseling [2]. Although guidelines and recommendations have been developed on the content of the medical information to include in antenatal counseling at the limits of premature viability [3,4], there is variation in how antenatal counseling skills are taught to neonatal-perinatal medicine (NPM) fellows in the United States, including whether fellows are trained to respond to families’ spiritual and emotional needs, whether fellows are trained to explain and provide comfort care at birth, and how often they have the opportunity to provide supervised antenatal counseling with real or simulated patients to facilitate feedback and learning [2,5,6]. Further, there are no currently available validated tools to evaluate antenatal counseling skills in NPM trainees.

Given the importance of antenatal counseling conversations, it is critical for NPM fellows to receive effective education on this topic from the outset of their training program. In the past, we have addressed this educational objective for junior fellows in our region through in-person interactive didactic sessions followed by simulated patient encounters designed to approximate realistic antenatal clinical situations. Simulated virtual antenatal counseling was also piloted by our group [7]. From years 2014 to 2019 antenatal counseling simulations were part of a two-day NPM multi-institutional fellow boot camp conducted at the beginning of the academic year. In addition to antenatal counseling skills, procedural skills and neonatal resuscitation were also taught using practice stations and simulation scenarios. Participants were comprised of first-year fellows and teaching faculty from three fellowship programs across the Pacific Northwest and Western Canada. 

As with nearly all other forms of medical education, our antenatal counseling curriculum in 2020 was threatened by the coronavirus disease 2019 (COVID-19) pandemic due to travel restrictions and physical distancing requirements. We improvised by conducting our annual regional NPM fellow boot camp virtually and maintained the day-long focused simulations on antenatal counseling. This technical report describes how we successfully taught NPM fellows from four fellowship programs a framework for antenatal counseling, including opportunities for simulated patient encounters, via video conference.

## Technical report

Preparation

Preparatory materials were shared electronically in advance of the session. Fellows were given articles on periviability counseling recommendations [3,4]. Faculty facilitators were given information on key learning points for each section of the didactic. There were three groups for the breakout sessions for counseling simulations, each with a different patient scenario. The faculty were preassigned to these breakout groups with one faculty to act as a parent and the other to facilitate the simulation and debriefing. Faculty who were acting as simulated parents were given detailed information on their parent character. To improve character consistency and generate more natural responses, acting parents were given information on their back story, medical literacy, thoughts on this pregnancy, hopes and fears, and some guidance on how to respond to potential things that could be said to them.

Example Scenario Provided to the Acting Parent

A 42-year-old G8P0 woman presents with a rupture of membranes at 22 weeks of gestation. This pregnancy has been complicated by hypertension, poor fetal growth, and a prior obstetric history of multiple miscarriages. The cervix is 3 cm dilated and the fetal feet are noted on cervical examination by the obstetrician. An ultrasound today estimates the fetus to weigh 390 g (fifth percentile). As the mother, this is your last viable embryo after multiple cycles of in vitro fertilization, and you are paralyzed with fear that the medical team will “give up” on your baby. Your partner is out of town and is already on a flight to get back to you. You feel alone since they can’t even join by phone while en route. You don’t want to wait as you feel the medical team is “dancing around what’s happening.” You have a moderate medical literacy as you have been through infertility treatment for many years, though you work in a non-medical field. You should remain standoffish until someone shows continued empathy for your situation. Whether your child has challenges or disabilities is not relevant to your decision-making and you are sensitive to how people discuss developmental issues since you work in special education.

Agenda and schedule

We used Zoom (San Jose, CA: Zoom Video Communications, Inc.) for video conferencing. In our educational sessions, participants joined individually from their homes or offices in Vancouver, British Columbia and the states of Washington, Oregon, and Hawaii. In part due to the virtual platform, we were able to expand the training to include participants from Hawaii. After initial introductions and orientation, we alternated brief interactive didactics with small group antenatal counseling simulations; the specific schedule is outlined in Table 1. The didactic sessions were held with the full group of participants, each led by a member of the teaching faculty. The simulations were held in break-out rooms with two faculty and two to three trainees per room, with one faculty in each breakout room acting as the expectant parent and the other as the facilitator. In the small group break-out rooms, the fellows observed one another in the simulation and every fellow had an opportunity to practice the skills for each break-out session. The day concluded with a wrap-up and reflections session. Fellows then continued on to an informal virtual meeting where they could debrief and network, while faculty debriefed separately on the session. The didactic teaching sessions were recorded for review by NPM fellows who were unable to attend the live boot camp day. However, the breakout simulation sessions were not recorded to create a confidential space for practicing these conversations.

**Table 1:** Antenatal counseling boot camp schedule

Agenda Item	Time Allotted
Introductions and goals	40 minutes
Zoom tutorial	5 minutes
Opening the visit/setting expectations	20 minutes didactic
	40 minutes breakout simulations
	20 minutes breakout debrief
Break	5 minutes
The headline	20 minutes didactic
	40 minutes group practice
Break for lunch	50 minutes
Empathic communication	20 minutes didactic
	30 minutes breakout simulations
	20 minutes breakout debrief
Break	5 minutes
Medical information	30 minutes didactic
Closing the visit	20 minutes didactic
	30 minutes breakout simulations
	20 minutes breakout debrief
Break	5 minutes
Wrap up	15 minutes full group
Fellow social hour	60 minutes
Faculty debrief	60 minutes

Learning content

Here, we will detail the key teaching points and strategies for each section of the day. The day was ordered according to how we recommend fellows organize their antenatal counseling patient visits from preparing for the consult through wrapping up. Based on the number of faculty and fellows in our group, we had three breakout groups. Each breakout group had its own case scenario and kept the same counseling storyline for the day. No two groups had the same case. Once in the breakout rooms, the fellows preparing to perform simulated counseling were given basic introductory information about the case such as the age and parity of the pregnant parent, gestational age, and the clinical concern.

Opening the Visit/Setting Expectations

The didactic portion of “setting the scene” focused on how the fellows will introduce themselves as well as information to gather in advance of meeting the patient and organizing the visit so that the key stakeholders are present. Participants volunteered to state how they introduce themselves and we discussed considerations for each. For example, many patients do not know what a neonatologist is but introducing yourself as a “baby doctor” can be interpreted as a student or intern to others, so fellows need to think about how best to explain their role. We also discussed who should be present for the consultation including the patient’s support person or partner, bedside nurse, and interpreter if needed. We also discussed how the concept of antenatal counseling can be introduced to patients and how to determine goals and set an agenda for the conversation, prioritizing the family’s needs and preferences. Table 2 summarizes the key information presented during the didactic session.

**Table 2:** Setting the scene elements

Element	Examples
Prepare by reviewing key medical and social history	Review obstetric history, ultrasound, and genetic testing results. Ascertain primary language, the urgency of consult, and the presence or whereabouts of support person(s). Ask the bedside nurse and family if this is a good time. “Is now an ok time to talk about your baby’s health?”
Set up the room to optimize communication	Obtain seats for all present and set them in a circle. Ensure correct people are present and troubleshoot missing people (e.g. join via speakerphone, reschedule). “Do we have all the important people here for your family or are we missing anyone?” Arrange interpreter as needed.
Frame the conversation to set expectations	Introduce team and ask to be introduced to family present. “We’d like to start by introducing ourselves, can you also share who you’ve brought today?” “I am here to talk about what would happen if your baby delivers early, what information are you hoping I will get to in our conversation? Many people prefer to hear the big picture, some prefer more detailed information, what works best for you?”

During the simulations in Zoom break-out rooms for this section, we focused on introductions and framing the conversation. In particular, it is helpful to have fellows practice how they will introduce themselves and how they will ask others present to introduce themselves to ensure everyone’s role is clear. Other important aspects of this part of the consult are determining how a patient prefers to receive information, asking permission to have a serious conversation, and setting goals for the conversation.

The Headline

The headline is the key statement clinicians want a patient to remember from their conversation [8]. Amidst the stress of preterm labor or a pregnancy complication, parents may not remember much of what was said during antenatal counseling so it is important to decide the headline prior to starting the visit and ensure it is clear, jargon-free, and accurately captures the key message. The headline should be delivered succinctly and clearly followed by a pause to allow the family to process the news, experience the emotions that come with difficult news, and clear their thoughts enough to hear more information. For this section of our virtual boot camp, we presented a brief didactic and then as a large group practiced creating headlines for a variety of bad news clinical scenarios that may occur in the NICU. An instructor would pose one clinical scenario, then call on a participant (teaching faculty or trainee) to attempt a headline. A second person would then propose a revision to that headline and so forth. Examples of clinical situations and corresponding headlines are provided in Table 3. Participants were encouraged to consider the wide variety of ways to word the same headlines and think about which felt best for their style of communication.

**Table 3:** Headline examples

Example Clinical Scenario	Potential Headline
Advanced preterm labor at 26 weeks gestation.	Your baby is coming very early; he will be very small and need to stay with us in the ICU for a long time.
New ultrasound finding of critical congenital heart disease at 34 weeks gestation	The ultrasound shows your baby’s heart did not form typically, and this will impact the medical care she needs at birth.
Hydrops fetalis at 25 weeks gestation	Your baby has extra fluid in many parts of his body and is unlikely to survive.
Pre-eclampsia with severe features at 32 weeks gestation	We expect your baby to survive and do well in the long term, but she will need to spend several weeks in the newborn ICU.

Empathic Communication

As neonatologists, empathic communication is important in all of our interactions with parents before and after their babies are born. We chose to teach about it in this order as emotion is typically elicited from delivering the headline. Therefore, it is particularly important to be equipped with empathic responses after delivering the headline. Further, when a parent is experiencing strong emotions until addressed they will not be in a mindset to effectively hear the medical information that follows. The didactic portion included asking trainees to brainstorm the emotions parents in these scenarios might be feeling. We defined empathy versus sympathy. We utilized elements of the VitalTalk® program (NURSE statements) on responses to emotion. NURSE is a VitalTalk acronym representing communication tools of naming the emotion (N), understanding (U), respecting the patient (R), supporting statements (S), and exploring questions (E) as shown in Table 4 [9]. During the simulation breakout session, the acting parents showed emotion and the trainees practiced acknowledging and responding to those emotions.

**Table 4:** NURSE is an acronym representing tools one may use to respond to emotion, as listed in the first column and adapted from VitalTalk®. The second and third columns contain examples specific to antenatal counseling. Source: Ref. [9]

Tool	Example 1	Example 2
Naming the emotion	I can hear how sad you are.	You seem frustrated about the plan for Sally.
Understanding	This is such a hard decision.	Any parent would be frustrated in this situation.
Respecting	You clearly love your baby and want him to not be in pain.	We want to continue talking to better understand your concerns.
Supporting	We are here to support you through these decisions.	The team is here to listen.
Exploring	Can you tell me more about what you hope will happen after he is born?	Tell me more about your concerns.

Medical Information

This section of the virtual boot camp was taught as an interactive didactic. We reviewed information to consider providing, with an emphasis on tailoring the content to the agenda established at the start of the visit and the family’s preferred method of receiving information. There is literature on recommended practices for content to include in antenatal counseling related to periviability and extreme prematurity. Prereading on this topic was assigned to the trainees to ensure they felt prepared to present relevant medical information [3,4]. Anecdotally, providing medical information is the area in which most physicians feel comfortable as compared with addressing emotions, so clinicians have a tendency to provide too much information as a way of staying in their comfort zone. We recommend limiting content to information that will help families understand the situation, prepare them for what to expect when their baby is born, answer their questions, explain how the NICU team will take care of their baby, and provide the necessary information for shared decision making if decisions are required. Current and former parents facing periviable birth or with a child in the NICU largely desire an active role in decision-making regarding their baby’s medical care [10,11]. We discussed how prematurity outcomes calculators such as the National Institute of Child Health and Human Development (NICHD) extremely preterm birth outcomes tool may be used for these consultations, but need to be interpreted with caution given the nuances of individual patients, local outcomes, and the limitations in the data itself [12]. For situations in which parents may choose comfort care only approach for their baby at birth, we reviewed the type of information to cover with an emphasis on supporting shared decisions, focusing on quality time with their dying newborn, addressing the newborn’s comfort [13], and knowing what specific palliative services and procedures are available at one’s institution (e.g., professional photographers, chaplains, cooling cots to preserve baby’s body and allow more time after death, and pediatric palliative care specialists) [3].

Table 5 includes content the neonatologist should be prepared to provide during the initial antenatal consultation. Fellows are encouraged to avoid medical jargon and acronyms (e.g., bronchopulmonary dysplasia {BPD}, intraventricular hemorrhage {IVH}, necrotizing enterocolitis {NEC}) and pause often to gauge whether a family is following the information or is overwhelmed and disengaged. Fellows are taught the signs of disengagement, such as lack of eye contact, silence, or flattened affect. Once a family disengages with information sharing, the conversation needs to shift back to focus on emotions and support or wrap up the visit to allow the family time to process. If time allows prior to the birth, a follow-up visit can be very helpful to complete the counseling and provide additional information.

**Table 5:** Key medical information* *Depends on clinical situation and family’s agenda and goals; in general, less is more.

Information Item	Example Wording
Prepare for the appearance of the baby	Your baby will be around 1lb and about the size of a soda can. Her skin will be very fragile looking.
Interventions and personnel expected right at birth	Our whole ICU team will come to your delivery and take care of your baby on a warmer bed in the corner of the room. Your baby will need to be covered with plastic to help keep her warm. All babies born this early need some help to breathe. She may need a tube to help her breathe.
Likely short term issues	Soon after she is born we will move her down to the special ICU for babies, called the NICU. She will be cared for in an incubator bed to stay warm, have a device on her face to help her breathe, and have an IV through her belly button or arm for fluids and medicines.
Approach to feeding	She will be too small to drink milk by mouth at first so we will give her nutrition with IV fluids and milk through a small feeding tube that goes from her nose to her stomach. If you are planning to provide breastmilk, we will help you learn how to pump and feed your milk through the tube.
Anticipated length of stay	Most babies need to stay in the NICU until around their original due date, some go home a little earlier and some stay for longer if they need to.
Risk for mortality and long term health outcomes	Unfortunately, some babies born this early do die. About 3 in 10 babies born as early as your baby live and get to go home. There are some long-term health issues for babies born early. Some need help to get around or eat food. There is no way to know right now how your baby will do, which is really hard.
Decisions they need to make, if any	Some babies are so sick when they are born that their heart rate is very low. If her heart is beating very slowly and isn’t improving with our care, this may mean she is too sick to survive. Some families would prefer they get to hold their baby if it looked like she was not going to survive, other families would prefer the team push on their baby’s chest to see if the heart will start beating again.
If comfort care only is considered: detail how comfort and support will be provided	In hearing about the risk of dying or having long-term health issues, some families choose comfort care for their babies. Comfort care is when we shift our focus away from invasive interventions and instead we help families spend quality time with their baby and help make sure their baby is not in pain before she dies. Is that something you might want to talk more about?

Closing the Visit

In the didactic portion of our "closing the visit" segment, we covered the teach-back method to check for patient understanding, emphasizing empathic communication and voicing support for the family in a difficult situation, and communication with other members of the healthcare team. Specifically, if a decision regarding initial care at birth such as full intensive care versus comfort care only has been made, it is critical to communicate that with the obstetric clinicians and document clearly in the chart. When time allows between initial neonatology consultation and the birth, we recommend neonatologists have a follow-up visit with the parents to answer any further questions they are likely to have. Table 6 summarizes key items with examples for concluding the visit. 

**Table 6:** Communication at the end of visit

Communication Tool	Example
Teach back	I shared a lot of information. To make sure I explained things well, would you mind telling me what you heard? Some people find it easiest to explain it back as they would to a family member who calls and asks what we talked about.
Empathic communication	This is such a hard situation, I am so sorry you and your family are going through this.
Support	Our team is in the hospital 24/7 and available whenever you need us.
Follow up	If you have any questions or want to talk more, just let your nurse know. They can find us day or night.
Care Coordination	Communicate any decisions made during the consultation with obstetric clinicians and document them in the chart.

Evaluations

Fellow performance was reviewed in the small group break-out rooms after each practice simulation using a structured observation form. Large group debriefing themes included identifying emotions expressed by the simulated parents, brainstorming methods of further exploring the emotions and values of the simulated parents, and sharing “headlines” that fellows used to convey key information during the simulations. Many fellows expressed limited previous experience with antenatal counseling and appreciated the dedicated time to learn this skill. Fears over mishandling these sensitive and high-impact conversations were common in participants. The six fellow participants from the 2020 course were invited to anonymously review the course electronically after it was completed. Three individual participants completed the evaluation which was a series of statements with a five-point Likert scale indicating the degree to which the respondent agreed with the statement and several free-text response questions to indicate something they learned in the course and overall comments about the course. All respondents either “agreed” or “strongly agreed” that they learned something during the course, that they enjoyed taking the course, and that the course improved their ability to conduct antenatal counseling. All respondents indicated the length of the course was “just right.” One participant provided a recommendation to change the course to allow for a full simulation at the end of the day to synthesize the components learned. In the evaluation, participants were also asked to rate the effectiveness of each individual faculty instructor; all of these evaluations were also positive with responses of either “excellent” or “very good.” These evaluations were comparable to the 2019 course which was held in-person. The 2019 evaluations used a four-point Likert scale and all participants rated the antenatal counseling components as either “good” or “excellent.”

## Discussion

In preCOVID years, we were able to teach antenatal counseling and communication skills in-person to junior NPM fellows using a combination of interactive didactic lectures and simulated counseling scenarios in a way that was perceived as effective by trainees. The current COVID-19 pandemic forced us to innovate our teaching. We used a similar teaching style over video conference and maintained smaller sections to ensure each component was taught and practiced before moving to the next section. The fellows were then able to focus on specific components of the antenatal counseling visit during small group break-out room simulations. This allowed us to teach a considerable amount of content in one day, organized in a way that maintained attention, and participation via a video conference platform. Fellow evaluations of the course showed they all agreed it was enjoyable, all agreed or strongly agreed it improved their ability to perform antenatal counseling, and all strongly agreed they learned something new. Respondents all reported the length of the course was just right including the antenatal counseling boot camp as well as social and networking time at the end of the day for fellows only without faculty present. The overall quality of the course was rated as “very good” or excellent” by all respondents. Free text evaluation comments included praise for breaking the consult down into sections to focus on individual components and communication skills.

The course detailed in this report could be improved with a couple of straightforward changes. For example, in the 2021 course, we plan to use skilled actors as expectant parents rather than using NPM faculty. This will allow all faculty to facilitate the debriefing sessions and help the simulated encounter to be more realistic. We will also use the same patient scenario for all small groups to improve the cohesiveness of the large group discussions and debriefings.

## Conclusions

This technical report details how antenatal counseling skills can be taught to junior NPM fellows via video conferencing by providing more traditional didactic lectures in addition to small group case simulations to practice the communication skills they have just learned. Given the high stakes of antenatal counseling for our patients and their families, providing this education and practice is critical. Our experience and the feedback from participants indicate that we can increase fellows’ perception of self-efficacy in antenatal counseling skills via videoconferencing. The use of a teleconference modality to provide this education enables necessary, timely training during a global pandemic. In the future, it will also increase accessibility for trainees who may be unable to travel to an educational session for a variety of reasons beyond a global pandemic such as personal medical conditions, financial cost, overlapping obligations, or visa limitations on international travel. As telemedicine becomes more widespread, counseling patients and families via video conference will continue to be an important skill for neonatologists in the future.

## References

[1] (2021). Premature birth. https://www.cdc.gov/reproductivehealth/features/premature-birth/index.html.

[2] Stokes TA, Watson KL, Boss RD (2014). Teaching antenatal counseling skills to neonatal providers. Semin Perinatol.

[3] Cummings J, Committee on Fetus and Newborn (2015). Antenatal counseling regarding resuscitation and intensive care before 25 weeks of gestation. Pediatrics.

[4] Geurtzen R, van Heijst AF, Draaisma JM (2019). Development of nationwide recommendations to support prenatal counseling in extreme prematurity. Pediatrics.

[5] Feltman DM, Williams DD, Carter BS (2017). How are neonatology fellows trained for antenatal periviability counseling?. Am J Perinatol.

[6] Arzuaga BH, Cummings CL (2016). Practices and education surrounding anticipated periviable deliveries among neonatal-perinatal medicine and maternal-fetal medicine fellowship programs. J Perinatol.

[7] Motz P, Gray M, Sawyer T, Kett J, Danforth D, Maicher K, Umoren R (2018). Virtual antenatal encounter and standardized simulation assessment (VANESSA): pilot study. JMIR Serious Games.

[8] (2021). Serious news. https://www.vitaltalk.org/guides/serious-news/.

[9] (2021). Responding to emotion: respecting. https://www.vitaltalk.org/guides/responding-to-emotion-respecting/.

[10] Edmonds BT, Savage TA, Kimura RE, Kilpatrick SJ, Kuppermann M, Grobman W, Kavanaugh K (2019). Prospective parents' perspectives on antenatal decision making for the anticipated birth of a periviable infant. J Matern Fetal Neonatal Med.

[11] Soltys F, Philpott-Streiff SE, Fuzzell L, Politi MC (2020). The importance of shared decision-making in the neonatal intensive care unit. J Perinatol.

[12] (2021). Extremely preterm birth outcomes tool. https://www.nichd.nih.gov/research/supported/EPBO/use.

[13] Garbi LR, Shah S, La Gamma EF (2016). Delivery room hospice. Acta Paediatr.

